# Evaluating the quality and readability of AI-generated information on adenomyosis: a comparative analysis of ChatGPT and deepseek regarding query-model consistency

**DOI:** 10.3389/fpubh.2026.1785229

**Published:** 2026-06-10

**Authors:** Ling Tian, Yan Wang, Ming-tao Yang, Hong-ni He, Tian-wen He, Ying Tang, Chuan Lin, Hui-quan Hu, Jun Li

**Affiliations:** 1 Department of Obstetrics and Gynecology, Beijing Anzhen Nanchong Hospital, Capital Medical University and Nanchong Central Hospital, Nanchong, Sichuan, China; 2 Department of Obstetrics and Gynecology, The Affiliated Nanchong Central Hospital of North Sichuan Medical College, Nanchong, Sichuan, China; 3 Department of Obstetrics and Gynecology, Women and Children's Hospital of Chongqing Medical University, Chongqing, China; 4 Department of Obstetrics and Gynecology, Chongqing Health Center for Women and Children, Chongqing, China; 5 Chongqing Research Center for Prevention & Control of Maternal and Child Diseases and Public Health, Chongqing, China; 6 Department of Obstetrics and Gynecology, Jinjiang District Maternal and Child Health Hospital of Chengdu, Chengdu, China

**Keywords:** adenomyosis, artificial intelligence, ChatGPT, deepseek, health information quality, patient education, readability

## Abstract

**Purpose:**

To systematically evaluate and compare the quality, readability, and query-model consistency of adenomyosis-related content generated by two large language models, ChatGPT (GPT-5) and DeepSeek (R1).

**Materials and methods:**

In total, 25 high-frequency patient queries were obtained based on Google Trends. Each query was processed using two interaction modes, namely, three consecutive repetitions and three independent cycles, on both large language models (ChatGPT GPT-5.0-web, released December 2025; DeepSeek R1-web, released November 2025). The generated texts (*n* = 300) were subsequently assessed for their readability [evaluated by Automated Readability Index (ARI), Flesch Reading Ease Score (FRES), and Gunning Fog Index (GFI)] and quality [assessed by DISCERN score, and Ensuring Quality Information for Patients (EQIP) tool]. Statistical comparisons were performed using non-parametric tests and *t*-tests.

**Results:**

In the cyclic mode, both ChatGPT and DeepSeek maintained stable output text readability and quality. DeepSeek-generated text demonstrated significantly superior readability across both interaction modes (lower ARI: 11.32 vs. 14.56, *p* < 0.001; higher FRES: 46 vs. 27, *p* < 0.001; lower GFI: 12.47 vs. 14.16, *p* < 0.001) and higher information quality (higher DISCERN: 62 vs. 43, *p* < 0.001; higher EQIP: 75 vs. 70, *p* < 0.001). Under the repetition mode, DeepSeek's output exhibited significant fluctuations across multiple metrics (ARI: *p* = 0.021; FRES: *p* = 0.015; GFI: *p* = 0.004; DISCERN: *p* = 0.013; EQIP: *p* < 0.001), while ChatGPT's output remained stable (all *p* > 0.05). Notably, the readability scores for both models indicated reading levels equivalent to undergraduate education, which is above the recommended level for general public health information.

**Conclusion:**

The findings of this study demonstrate that when generating information on adenomyosis, DeepSeek outperforms ChatGPT in terms of readability and several information quality metrics, whereas ChatGPT exhibits greater consistency in its outputs. However, the reading difficulty of texts generated by both models exceeds the level suitable for the general public, representing a key practical constraint limiting direct public use. Based on these results, AI chatbots may serve as complementary tools in patient education; however, their outputs should undergo expert review and be optimized for comprehensibility before broader clinical application. For clinicians and patients, these findings emphasize the importance of critically appraising AI-generated information and using it as a supplement to, rather than a substitute for, professional medical consultation.

## 1 Introduction

As a common benign gynecological disease, adenomyosis is a condition where endometrial glands and stroma invade in the myometrium, and the surrounding muscle layer thickens (1). This condition shows diversified clinical manifestations, primarily including progressively deteriorating dysmenorrhea, menorrhagia, chronic pelvic pain, and infertility, seriously threatening the quality of life and fertility among women of reproductive age (2, 3). In recent years, with the advancements in imaging diagnostic technologies, in particular the widespread application of transvaginal ultrasound and magnetic resonance imaging (MRI), the adenomyosis diagnosis rate in young and infertile women has significantly increased, and its status is an important public health issue (4, 5).

However, the etiology of adenomyosis is complex, which involves various mechanisms such as genetic susceptibility, epigenetic alterations, local estrogen dominance, progesterone resistance, and chronic inflammation, and it remains challenging to manage this condition in clinical practice, especially for patients with fertility aspirations (6, 7). Patients often require accurate and comprehensible information regarding the disease diagnosis, treatment options (like pharmacological therapy, gonadotropin-releasing hormone (GnRH) agonist pretreatment, and conservative surgery), and the influence on assisted reproductive technology outcomes (8, 9). As the Internet technology develops rapidly, the web is becoming the crucial approach to obtain medical information by the public (10). Users are allowed to access massive disease-associated content through diverse online platforms (11), which increases the health issue awareness among them and promotes health education initiatives as well as personalized medical decision-making (12). In the context of uneven medical resource distribution, web-based platforms are the specific approaches for bridging information gaps (13). Against this backdrop, the Internet and artificial intelligence (AI) have become vital channels for patients to seek health information.

Large language models have demonstrated significant application potential in the field of medical information generation recently. For instance, models such as ChatGPT and DeepSeek can provide immediate, easily accessible health consultations, which may potentially address gaps in traditional patient education (14, 15). However, the quality (such as the accuracy, comprehensiveness, and reliability) and readability (namely, the ease of understanding for the target audience) of the content generated by these models are key determinants of their clinical applicability. As noted in existing studies, while AI models can provide structured information in other medical domains (such as bariatric surgery), their reliability and readability can vary, and they usually lack necessary humanistic considerations (16). More importantly, considering the complexity of medical information, the content generated by AI models should be not only scientifically accurate, but also consistent with professional clinical guidelines and adaptable to the needs of patients with varying health literacy levels. In recent years, there has been growing interest in the application of artificial intelligence (AI) in gynecological disease management, particularly in areas such as imaging diagnosis, disease prediction, and patient education. For example, Cetera et al. (17) reviewed the potential and challenges of using AI in the management of endometriosis and adenomyosis, highlighting both its opportunities and risks as a clinical decision-support tool. Despite these advances, systematic and standardized comparative studies evaluating the quality and readability of AI-generated patient-facing health information on adenomyosis remain scarce. To address this gap, the present study systemically assesses and compares the readability, information quality, and consistency of responses generated by two mainstream large language models in response to common patient inquiries about adenomyosis.

Accordingly, this study conducts an exploratory comparative analysis of the performance of two representative large language models—ChatGPT (GPT-5) and DeepSeek (R1)—in answering a predefined set of clinical questions related to adenomyosis. The objective is to preliminarily evaluate and compare the performance of both models in specific tasks, including overall information quality, reliability, readability, and stability of the generated text content.

## 2 Material and methods

### 2.1 Study design

This study conducted a comparative evaluation of the performance of ChatGPT and DeepSeek in responding to patient-centered questions related to adenomyosis. For analyzing the adenomyosis-related search trends, we utilized the Google Trends platform (https://trends.google.com/) for identifying adenomyosis-related questions with the highest search frequency between January 2004 and September 2025. All interactions with the AI models were conducted in September 2025, using the following publicly available versions: ChatGPT (GPT-5.0-web, released December 2024) and DeepSeek (DeepSeek-R1-web, released November 2024). The configuration settings, including DeepSeek's default “deep thinking” mode, remained consistent throughout the testing period.

**Figure 1:**
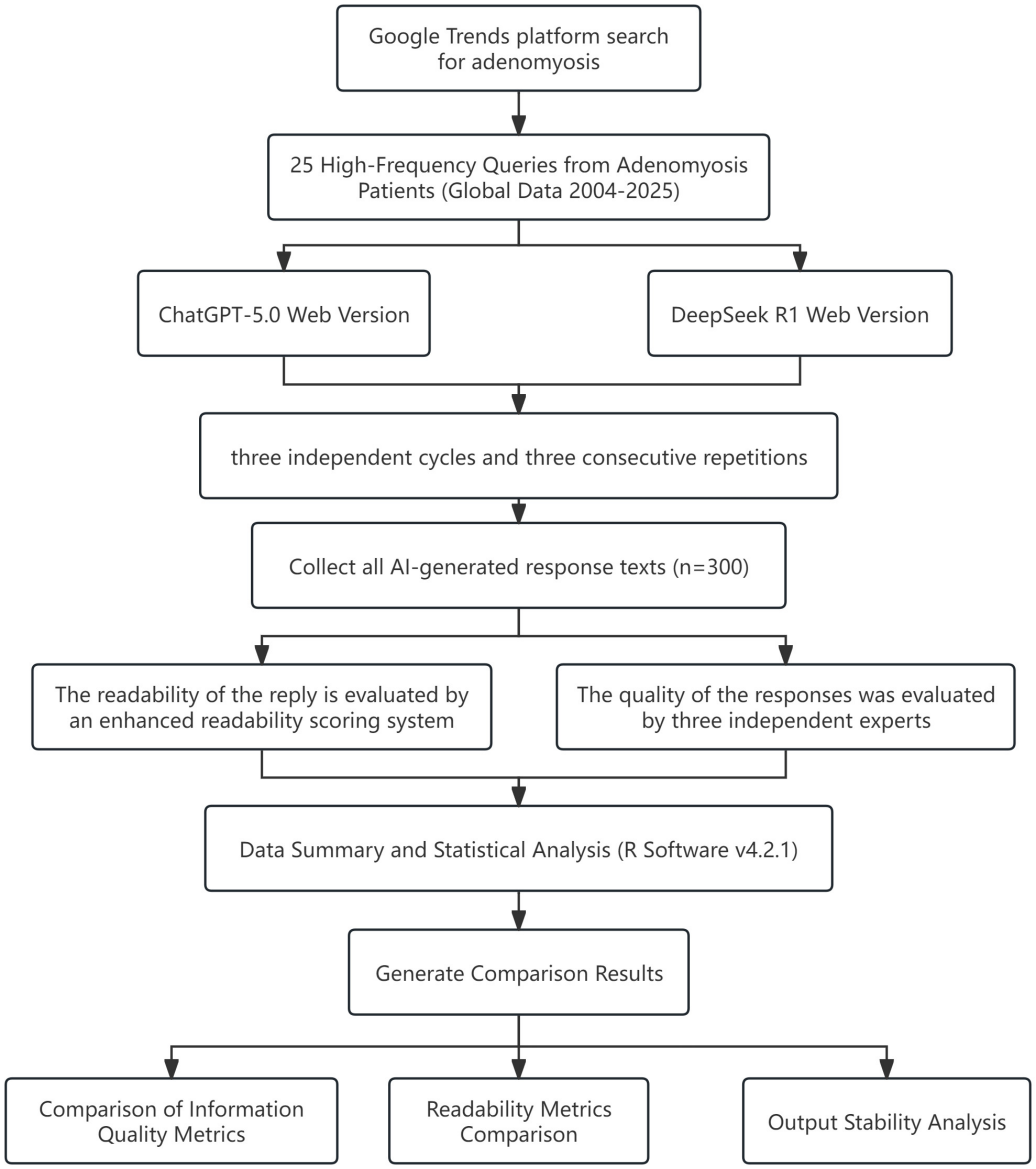
Flow chart of question inclusion and response evaluation.

In this search, we set the geographical setting as “global” (18). Subsequently, the 25 most frequently searched questions were selected based on their relevance to the topic. Non-English search terms were translated into English to ensure consistency in prompt language used for both models. Two authors independently screened the full list of adenomyosis-related search terms retrieved from Google Trends, applying predefined inclusion and exclusion criteria. Any disagreements were resolved through discussion with a third author, and the final set of queries was approved by consensus. To be included, search terms had to directly address patient information needs regarding adenomyosis, specifically its definition, symptoms, causes, treatment, and impact on fertility. Exact duplicate terms were excluded. Afterwards, the 25 high-frequency queries obtained based on Google Trends were examined with two interaction modes of ChatGPT-5.0 Web Version and DeepSeek R1 Web Version. The first interaction mode, called “repetition mode,” posed the same question thrice consecutively for assessing whether the responses of ChatGPT were consistent in short intervals. The second one was “cyclic mode,” which required sequentially asking several questions relevant to one topic, and repeating across three cycles. For mitigating the possible influence or memory bias induced by user-specific prompts, we performed strict controls in testing. Besides, the device and login environment were kept the same during the queries, thereby eliminating variability resulting from hardware and account-specific factors. All queries were executed using a newly created, untrained account. Under the repetition mode, each question was repeatedly asked thrice in one chat window, after which, the conversation history was cleared prior to conducting a new query. In the cyclic mode, one individual cycle of relevant questions was performed in a chat window, and then the conversation history was eliminated prior to the initiation of the next cycle, thus ensuring that every interaction was independent and the interference caused by the model's contextual memory was minimized. Flow chart of question inclusion and response evaluation in Figure 1.

### 2.2 Ethical approval

This study did not involve any human subjects, patient data, or clinical interventions. It constitutes a secondary analysis of AI-generated content and does not involve any identifiable personal information; therefore, ethical approval and informed consent were not required.

### 2.3 Assessment of answer readability and quality

The quality and readability of the generated text was evaluated by numerous established metrics, such as Automated Readability Index (ARI), Flesch Reading Ease Score (FRES), Gunning Fog Index (GFI), DISCERN score, and Ensuring Quality Information for Patients (EQIP).

The ARI, proposed by the linguists E. A. Smith and R. J. Senter, is a metric for gauging linguistic complexity and reading difficulty, which indicates the educational level necessary for comprehending a text (19). For instance, when the ARI score is 6.2, it corresponds to a sixth-grade reading level, while that of 12.2 suggests that the text is suitable for college graduates. A greater ARI score indicates a greater reading difficulty, signifying that this text may be more suitable for an audience with a higher educational level. In contrast, a lower score reflects simpler linguistic structures, making the text comprehensible for ordinary readers. In the ARI formula, the average number of characters (including any letters, numbers, symbols, etc., excluding spaces between characters) per word and the mean word count in each sentence are taken into account. The ARI score is determined according to the overall character, word and sentence counts.

The FRES, which was proposed by the American linguist Rudolf Flesch in 1948, is an early and extensively utilized tool to assess text readability (20). The simplicity of its formula has greatly contributed to its popularity across many writing disciplines. The score is usually adopted in the medical area for evaluating whether scientific information, medication leaflets and health guidelines are readable, thus guaranteeing the accessibility and comprehensibility of these texts for the public. The scale ranges from 0 to 100, with a higher score indicating greater readability. Generally, texts scoring 90–100 points are considered very easy to read, while scores below 30 points suggest that a text is at a challenging, academic level.

GFI is another tool used to assess text readability (21). This index calculates two factors: sentence length (with a shorter sentence being easier to understand) and the number of complex words (referring to words with at least three syllables). For example, a GFI score of 9 indicates that a 9th-grade student can comprehend the text, while a score of 12 corresponds to a high school reading level, and a score above 17 signifies the comprehension level of a college graduate.

The DISCERN score can objectively and systemically assess the scientific quality and usefulness of medical information. Initially designed by the Department of Public Health and Primary Care in the University of Oxford (22), the DISCERN instrument covers 16 items (each scored from 1 to 5 points) that are grouped in three domains: the reliability of the information, the quality of the information on treatment choice, and the overall quality. A high score (>55 points) indicates high-quality information and a reliable source. A moderate score (39–54 points) suggests acceptable quality, but with potential significant omissions or shortcomings, which warrants cautious use. A low score (< 39 points) reflects poor-quality information with serious flaws, which may be misleading and is thereby not recommended as a basis for decision-making.

The Ensuring Quality Information for Patients (EQIP) comprises 20 items (23). In line with the EQIP standards, the scores of 1, 0.5 and 0 are awarded for full, partial and no compliance, respectively. The eventual score is represented by a percentage and calculated by (Total EQIP Score / 20) ^*^ 100%. A high score (e.g., > 75%) suggests high-quality information regarding the content, design, transparency, and usability. A moderate score (e.g., 50%−75%) suggests acceptable information quality with several areas to be improved. A low score (< 50%) reflects poor information quality that may be misleading, difficult to use, or lacking critical details.

To be specific, ARI, FRES and GFI indices of each generated text were calculated by an enhanced readability scoring system (https://readabilityformulas.com/readability-scoring-system.php). The three evaluation experts invited for this study are all practicing gynecologists with senior professional titles. Each has over 10 years of clinical experience in obstetrics and gynecology, with expertise encompassing the diagnosis and treatment of benign gynecological conditions, including adenomyosis. They are affiliated with three different Grade A tertiary teaching hospitals in China, located in distinct cities (Shanghai, Chongqing, and Nanchong). None of these experts were involved in the design or data collection of this study, ensuring the independence of the evaluation process. Experts were selected through purposive sampling based on explicitly defined inclusion criteria: (1) gynecologists holding a senior professional title; (2) possessing extensive clinical experience in general gynecology, with particular familiarity in the diagnosis and management of adenomyosis; and (3) agreeing to and committing to complete the review of all assigned texts in accordance with the study protocol. Throughout the scoring process, the experts were completely blinded to the model that generated each text. To minimize assessment bias, the following measures were implemented: prior to evaluation, all experts received unified training via an online meeting on each scoring criterion of the DISCERN and EQIP instruments. They practiced using a set of non-study samples until reaching consensus on the scoring standards. Each expert then completed the scoring of all texts independently in an undisturbed environment. The final score for each assessment item was calculated as the average of the three experts' ratings.

### 2.4 Statistical analysis

R software (version 4.2.1) was adopted for statistical analysis. Continuous data were first evaluated by Shapiro–Wilk test for normality. Thereafter, non-normally-distributed data were presented as median with interquartile range (Q1, Q3) and compared by Kruskal–Wallis rank sum test among several groups or by Wilcoxon rank sum test between groups. While normally-distributed data were represented by mean ± standard deviation and compared through one-way ANOVA or Welch's t-test. *p* < 0.05 (two-tailed) suggested statistical significance.

## 3 Results

### 3.1 Issues related to adenomyosis

**Figure 2:**
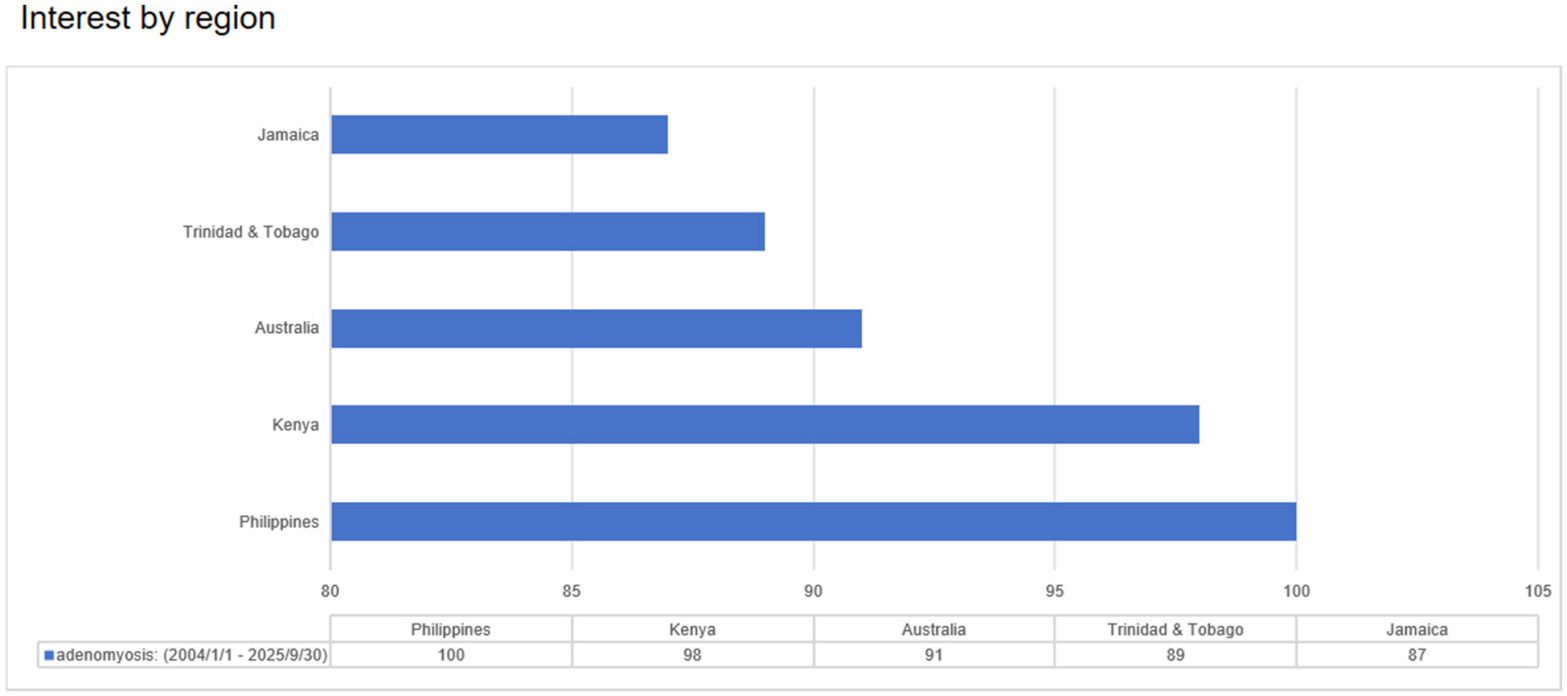
Global Distribution of Popular Searches for Adenomyosis by Region, January 2004–September 2025. Data source: Google Trends (https://www.google.com/trends).

**Figure 3:**
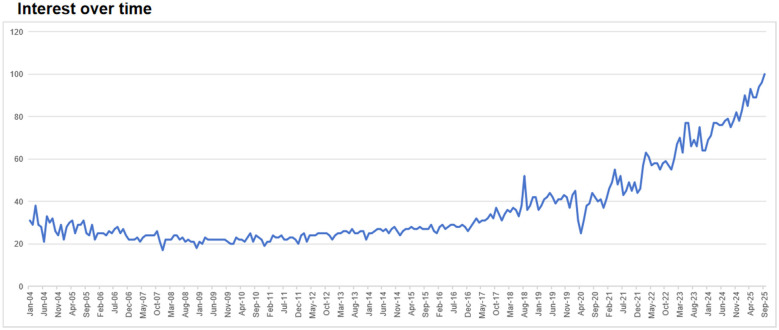
From January 2004 to September 2025, the search popularity for the term “adenomyosis.” Data source: Google Trends (https://www.google.com/trends).

As revealed by analysis of Google Trends data, the globally most-searched terms related to adenomyosis are “adenomyosis endometriosis,” “adenomyosis uterus,” and “uterus,” with the relative search interest scores of 100, 98, and 96, respectively. In addition, other search terms encompassed various topics, like symptoms and root causes of adenomyosis, and the possible treatments (Table 1). A global search distribution map illustrated regions with the most high-frequency search of the term “adenomyosis”. It was clearly observed that countries with significant volumes of searches of information on “adenomyosis” included Cuba, Italy, and Venezuela, among others (Figure 2). Furthermore, according to the temporal trend analysis, the global search interest for “adenomyosis” was always high since 2004 (Figure 3).

**Table 1:** Google trends data for the top 25 exact prompts with the highest search volume on adenomyosis globally from January 2004–September 2025.

Rank	Exact prompts/keywords	Relevance
1	Adenomyosis endometriosis	100
2	Adenomyosis uterus	98
3	Uterus	96
4	Endometriosis	96
5	Adenomyosis symptoms	54
6	Adenomyosis pain	52
7	Adenomyosis treatment	48
8	Uterine adenomyosis	48
9	Adenomyosis meaning	41
10	What is adenomyosis	40
11	Adenomyosis of uterus	39
12	Adenomyosis hysterectomy	28
13	Adenomyosis ultrasound	28
14	Hysterectomy	27
15	Adenomyosis and endometriosis	27
16	Adenomyosis pregnancy	25
17	Fibroids	23
18	Adenomyosis in uterus	22
19	Adenomyosis	19
20	Fibroid	19
21	Adenomyosis vs. Endometriosis	19
22	Endometriosis vs. Adenomyosis	19
23	Adenomyosis causes	18
24	Endometrium	16
25	Adenomyosis uteri	14

Global search data are presented alongside comparative data from the United States and China (Table 2). In U.S.-based searches, the most frequent terms related to adenomyosis include “endometriosis,” “endometriosis adenomyosis,” and “uterus adenomyosis,” with the relative search interest scores of 100, 99, and 93, respectively. In China-based searches, the high-frequency terms are “endometriosis,” “endometriosis,” and “menorrhagia,” with the relative scores of 100, 51, and 48, respectively. It should be noted that, Google is not the predominant search engine in China, therefore, insights derived from the China region are likely limited and should be interpreted with caution.

**Table 2:** Relative search interest rankings for adenomyosis across global, United States, and Chinese geographic scales from January 2004–September 2025.

	Global		United States		China	
	Query	Search interest	Query	Search interest	Query	Search interest
1	adenomyosis endometriosis	100	endometriosis	100	endometriosis	100
2	adenomyosis uterus	98	endometriosis adenomyosis	99	menorrhagia	51
3	uterus	96	uterus adenomyosis	93	adenomyosis treatment	48
4	endometriosis	96	adenomyosis endometriosis	93	adenomyosis Chinese	31
5	adenomyosis symptoms	54	uterus	92	adenomyosis definition	28
6	adenomyosis pain	52	adenomyosis uterus	91	adenomyosis symptoms	20
7	adenomyosis treatment	48	adenomyosis symptoms	61	dysmenorrhea	20
8	uterine adenomyosis	48	uterine adenomyosis	56	adenomyosis meaning	0
9	adenomyosis meaning	41	adenomyosis pain	52		
10	what is adenomyosis	40	what is adenomyosis	46		
11	adenomyosis of uterus	39	hysterectomy	37		
12	adenomyosis hysterectomy	28	adenomyosis hysterectomy	37		
13	adenomyosis ultrasound	28	adenomyosis treatment	31		
14	hysterectomy	27	adenomyosis of uterus	31		
15	adenomyosis and endometriosis	27	adenomyosis ultrasound	28		
16	adenomyosis pregnancy	25	fibroids	27		
17	fibroids	23	adenomyosis and endometriosis	25		
18	adenomyosis in uterus	22	endometriosis and adenomyosis	25		
19	icd 10 adenomyosis	19	endometriosis vs adenomyosis	22		
20	fibroid	19	adenomyosis vs endometriosis	22		
21	adenomyosis vs endometriosis	19	adenomyosis pregnancy	21		
22	endometriosis vs adenomyosis	19	adenomyosis causes	16		
23	adenomyosis causes	18	adenomyosis of the uterus	15		
24	endometrium	16	endometrium	15		
25	adenomyosis uteri	14	icd 10 adenomyosis	15		

### 3.2 Readability and quality stability comparison in repetition mode

Table 3 compares the readability and quality of the texts produced by DeepSeek and ChatGPT under a repetition strategy where each query was submitted thrice consecutively. For DeepSeek, there were significant differences in ARI (*p* = 0.021), FRES (*p* = 0.015), GFI (*p* = 0.004), DISCERN (*p* = 0.013), and EQIP (*p* < 0.001) across the three repetitions, exhibiting variability in text quality when repeated words were used. In contrast, ChatGPT showed no significant differences in any metric (all *p* > 0.05) across repetitions, suggesting its greater consistency in output under repetitions.

**Table 3:** Readability and quality stability comparison in repetition mode.

Characteristic	Deepseek *N* = 75				ChatGPT *N* = 75			
	Repeat (1) *N* = 25	Repeat (2) *N* = 25	Repeat (3) *N* = 25	*p*-value	Repeat (1) *N* = 25	Repeat (2) *N* = 25	Repeat (3) *N* = 25	*p*-value
**ARI, Median (Q1, Q3)**	11.76 (11.02, 13.08)	11.23 (10.26, 12.07)	10.90 (9.85, 11.79)	0.021^&^	14.59 (13.20, 15.96)	14.62 (13.43, 15.50)	14.75 (12.87, 15.06)	0.999^&^
**FRES, Median (Q1, Q3)**	41 (36, 45)	47 (41, 51)	46 (44, 51)	0.015^&^	29 (26, 40)	28 (22, 36)	27 (20, 37)	0.743^&^
**GFI, Mean** **±SD**	12.96 ± 1.23	11.98 ± 1.51	11.70 ± 1.33	0.004^*^	14.60 ± 1.71	14.52 ± 1.86	14.49 ± 1.85	0.972^*^
**DISCERN, Median (Q1, Q3)**	65 (55, 73)	59 (55, 66)	53 (49, 63)	0.013^&^	43 (40, 47)	46 (45, 49)	48 (45, 49)	0.077^&^
**EQIP, Median (Q1, Q3)**	85 (78, 90)	80 (70, 85)	75 (65, 80)	< 0.001^&^	58 (48, 65)	61 (59, 66)	63 (58, 65)	0.243^&^

^&^Kruskal-Wallis rank sum test; ^*^One-way analysis of means.

### 3.3 Readability and quality stability comparison in cyclic mode

Table 4 presents the results using a three-cycle generation approach (full-text regeneration). For DeepSeek, significant difference was only detected in FRES across diverse cycles (*p* = 0.019), while the other metrics remained stable. ChatGPT again demonstrated no significant differences in any measure (all *p* > 0.05) across different cycles, reinforcing its consistency in cyclic generation. To provide a holistic comparison, data from both modes were aggregated to examine the overall model performance of DeepSeek vs. ChatGPT.

**Table 4:** Readability and quality stability comparison in cyclic mode.

Characteristic	Deepseek *N* = 75				ChatGPT *N* = 75			
	Cycle (1) *N* = 25	Cycle (2) *N* = 25	Cycle(3) *N* = 25	*p*-value^1^	Cycle (1) *N* = 25	Cycle (2) *N* = 25	Cycle(3) *N* = 25	*p*-value^1^
**ARI, Median (Q1, Q3)**	11.09 (10.66, 11.53)	11.32 (10.40, 12.06)	11.71 (10.91, 12.20)	0.170	14.46 (13.64, 15.79)	14.55 (13.20, 15.48)	14.80 (14.16, 16.03)	0.504
**FRES, Median (Q1, Q3)**	48 (44, 52)	47 (41, 51)	42 (38, 45)	0.019	28 (24, 31)	27 (24, 33)	27 (24, 32)	0.874
**GFI, Median (Q1, Q3)**	12.40 (11.10, 13.10)	12.50 (11.20, 13.30)	12.70 (12.00, 13.40)	0.317	14.50 (13.80, 14.70)	14.20 (13.60, 15.10)	14.40 (13.60, 15.10)	0.941
**DISCERN, Median (Q1, Q3)**	61 (42, 72)	62 (36, 73)	62 (50, 72)	0.954	41 (37, 49)	43 (39, 51)	43 (39, 52)	0.603
**EQIP, Median (Q1, Q3)**	75 (70, 93)	75 (58, 93)	75 (70, 90)	0.908	68 (58, 73)	70 (68, 75)	73 (63, 75)	0.422

^1^Kruskal-Wallis rank sum test.

### 3.4 Compare the readability and quality of text generated by ChatGPT and DeepSeek under both modes

Table 5 summarizes the data obtained under both repetition and cyclic conditions. In the repetition mode, compared to ChatGPT, the text generated by DeepSeek demonstrated significantly superior readability (lower ARI: 11.27 vs. 14.69, *p* < 0.001; higher FRES: 45 vs. 28, *p* < 0.001; lower GFI: 12.21 vs. 14.54, *p* < 0.001) and higher information quality (higher DISCERN: 58 vs. 45, *p* < 0.001; higher EQIP: 80 vs. 60, *p* < 0.001) in both interaction modes. In the cyclic mode, the text generated by DeepSeek showed significantly superior readability (lower ARI: 11.32 vs. 14.56, *p* < 0.001; higher FRES: 46 vs. 27, *p* < 0.001; lower GFI: 12.47 vs. 14.16, *p* < 0.001) and higher information quality (higher DISCERN: 62 vs. 43, *p* < 0.001; higher EQIP: 75 vs. 70, *p* < 0.001) across both interaction modes than those of ChatGPT. A systematic analysis was conducted on the sub-score items closely related to accuracy within the EQIP and DISCERN tools. Detailed results are presented in Supplementary Table S1.

**Table 5:** Compare the readability and quality of text generated by ChatGPT and DeepSeek under both modes.

Characteristic	Repeat *N* = 150			Cycle *N* = 150		
	Deepseek *N* = 75	ChatGPT *N* = 75	*p*-value	Deepseek *N* = 75	ChatGPT *N* = 75	*p*-value
**ARI, Median (Q1, Q3)**	11.27 (10.39, 12.18)	14.69 (13.20, 15.50)	< 0.001^#^	11.32 (10.79, 12.12)	14.56 (13.64, 15.83)	< 0.001^#^
**FRES, Median (Q1, Q3)**	45 (39, 50)	28 (21, 38)	< 0.001^#^	46 (41, 51)	27 (24, 33)	< 0.001^#^
**GFI, Mean** **±SD**	12.21 ± 1.45	14.54 ± 1.78	< 0.001^$^	12.47 ± 1.24	14.16 ± 1.21	< 0.001^$^
**DISCERN, Median (Q1, Q3)**	58 (53, 68)	45 (41, 49)	< 0.001^#^	62 (36, 72)	43 (38, 52)	< 0.001^#^
**EQIP, Median (Q1, Q3)**	80 (73, 85)	60 (53, 65)	< 0.001^#^	75 (70, 93)	70 (63, 75)	< 0.001^#^

^#^Wilcoxon rank sum test; ^$^Welch Two Sample *t*-test.

## 4 Discussion

In this study, we systematically evaluated the quality, readability, and consistency of adenomyosis-related texts generated by two mainstream large language models—ChatGPT (GPT-5) and DeepSeek (R1). The results indicated that DeepSeek significantly outperformed ChatGPT in terms of the readability metrics (such as ARI and GFI) and achieved higher scores in information quality assessments (DISCERN and EQIP), suggesting its greater potential applicability in patient education. Notably, some of the metrics of DeepSeek exhibited variability in the repetition mode, whereas ChatGPT demonstrated stronger consistency, reflecting certain differences in the design of response stability between the two models. This study provides empirical data for assessing the performance of large language models in generating patient education information for a specific gynecological condition—adenomyosis. While prior research has explored the role of AI in the management of endometriosis and adenomyosis from a broader perspective, detailed assessments specifically focusing on the quality and readability of textual information—the primary interface directly encountered by patients—remain limited (17). Our findings indicate that in the task of generating patient-directed text information, different models exhibit significant variations in quality, readability, and stability. These results offer actionable insights for the future development of more reliable AI-assisted health education tools.

Adenomyosis, as a chronic gynecological disease with complex etiology and diverse clinical manifestations, creates an urgent need for patients to obtain information on its diagnosis, treatment, and impact on fertility (24, 25). The Internet and AI tools have become important channels for patients to access health information, yet the accuracy and comprehensibility of the generated content will directly affect clinical decision-making and patient psychology (26, 27). As previously suggested, the stability of medical information regarding various diseases generated by ChatGPT is satisfactory (28, 29). Consistent with these earlier findings, our study further confirmed that ChatGPT demonstrated good response stability when providing information on multiple diseases (14, 15, 19, 30). This can be associated with optimization capabilities in natural language processing and stable performance of large language models in training on massive datasets, providing evidence supporting their application in patient health information acquisition and medical health education. In contrast, the observed variability in DeepSeek's outputs under the repetition mode may reflect underlying differences in how the model processes deterministic, repeated prompts compared to ChatGPT. Although architectural distinctions or variations in training protocols, such as reinforcement learning strategies, contribute to this behavior, further technical investigation is required to clarify the underlying mechanisms (28).

Readability remains a key concern for AI-generated medical information (28, 31). We assessed the reading difficulty of ChatGPT- and DeepSeek-generated texts by metrics ARI, FRES, and GFI scores, and our obtained outcomes were consistent with prior research (32, 33). The information on adenomyosis obtained from the two models required an educational level equivalent to an undergraduate student, indicating the overly complex language. Therefore, individuals with limited health literacy may struggle to fully grasp key concepts related to their conditions, treatment options, and potential risks, thereby limiting the practical utility of such information for the general public (34, 35). Considering the diverse medical knowledge and educational levels of patients with adenomyosis, high readability barriers may hinder their comprehension of the generated content. This goes against the goal of more accessible medical information dissemination and may exacerbate concerns about the disease (36, 37). When recommending or discussing AI-generated information with patients, clinicians should recognize that raw model outputs may fall outside patients' reading comfort zone. During consultations, it may be necessary for clinicians to proactively paraphrase, simplify, and contextualize the AI-generated content. These findings also suggest that current AI tools, in their default configurations, are not yet optimized for direct patient use without professional intermediary adaptation.

Therefore, it is necessary to generate content suitable for readers with average reading skills. Developing personalized medical information at varying levels of complexity—through iterative model training or prompt engineering—tailored to patients' specific needs and cultural-literacy background represents a promising direction for future research and application. Although the two models should be further improved to meet the health literacy standards, it is noteworthy that the slightly higher ease-of-understanding scores and the lower grade level of DeepSeek suggest that it may lay more emphasis on readability than ChatGPT.

DISCERN and EQIP are instruments designed to help patients and health information users assess the quality of written materials concerning treatment options (38). AI language models can assist patients in understanding treatment-related information and expectations, thereby enabling them to choose the most suitable options. This capability holds significant value for individuals and organizations involved in using or developing information to support treatment decision-making. However, other studies demonstrate that there are still significant limitations of ChatGPT in the quality and reliability of its generated medical information about various diseases (14, 39). A similar conclusion is drawn in this study: ChatGPT's DISCERN and EQIP scores fall within the moderate range (45 and 60 points, respectively, in the repeated questioning mode), indicating acceptable but improvable information quality. The significant differences in DISCERN and EQIP scores across various models also show practical implications for patient education. Through expert evaluation, two specific patterns of relative weakness in ChatGPT's responses are identified: (1). Insufficient comprehensiveness and depth of information: When addressing queries involving comparisons of treatment options, ChatGPT's responses tend to simply list conventional options (such as painkillers, hormone therapy, hysterectomy), without providing detailed elaboration on the respective treatment plans. In responses concerning “impact on fertility,” the content frequently remains at a general level (like “may cause difficulty in conceiving”), lacking in-depth discussion of the underlying pathological mechanisms, quantitative data on assisted reproductive technology (ART) success rates, and staged management strategies (including GnRH agonist pretreatment). This lack of information may fail to meet the deep informational needs of users, particularly younger patients with strong fertility intentions, thereby limiting their ability to make fully informed decisions (2). Lack of transparency in information sources and timeliness alerts: This is an important aspect emphasized by tools such as DISCERN and EQIP. ChatGPT's responses almost never proactively cite specific clinical guidelines (like FIGO or ACOG guidelines), peer-review literature, or indicate the information update date. Although its internal knowledge may include information up to a certain date, this is not explicitly communicated to users. The absence of source attribution and update declarations undermines the credibility and verifiability of information, leaving users unable to assess whether the information reflects current evidence. The detailed item comparison in Supplementary Table S1 further elucidates these patterns, indicating that ChatGPT's generated materials require further optimization on items related to therapeutic benefits, risks, and shared decision-making support.

The superior performance of DeepSeek-R1 in readability and quality scoring likely stems from its deep reasoning mechanism. As Guo et al. (40) demonstrated, DeepSeek-R1′s reasoning capabilities were encouraged through a pure reinforcement learning framework, which fostered the emergence of advanced reasoning patterns such as self-reflection, verification, and dynamic strategy adaptation. This chain-of-thought reasoning, which generates step-by-step thinking before arriving at a final answer (41), appears to promote outputs that are more structured, logically coherent, and reader-friendly. By decomposing complex medical concepts into sequential reasoning steps, this mechanism reduces sentence-level information density and improves textual coherence—both cognitive factors that are especially beneficial for readers with limited domain knowledge (42). These features align with our quantitative findings of lower ARI and GFI scores and higher FRES and DISCERN ratings for DeepSeek-generated texts. Supporting this interpretation, Zhou et al. reported that DeepSeek-R1 produced the most readable responses among four evaluated models when generating patient education materials for spinal surgery, with FKGL scores ranging from 7.2 to 9.0 (43). Similarly, Zang et al. found that DeepSeek-R1 achieved the highest overall content quality on the DISCERN-AI scale in patient education materials for gynecomastia (44).

The greater output consistency observed with ChatGPT may be attributed to its training paradigm, particularly the intensive use of Reinforcement Learning from Human Feedback (RLHF), which prioritizes response stability, safety, and adherence to a narrower range of acceptable outputs. Ouyang et al. showed that RLHF-based instruction tuning aligned model outputs more closely with human preferences, emphasizing reliability and harmlessness (45). Bai et al. (46) further demonstrated that RLHF could be systematically applied to balance helpfulness and safety in conversational AI systems. This optimization for consistency and user satisfaction likely reduces variability across repeated queries, as reflected in our stability analysis. Alfertshofer et al. provided direct empirical evidence supporting this phenomenon, showing that ChatGPT-4 responded with significantly greater consistency than ChatGPT-3.5 when answering 450 medical examination questions across repeated entries (47). Similarly, Meyer et al. (48) discovered that newer ChatGPT versions (GPT-4, GPT-4o) exhibited markedly less variability in repeated reference-interval outputs compared to earlier versions. Mechanistically, a recent diagnostic study described how overly constrained RLHF topologies could lead to narrow latent attractor basins, resulting in reduced token entropy and a tendency toward repetitive outputs—a phenomenon termed “RLHF-induced rigidity” (49). While such rigidity may enhance stability, it also limits the model's capacity for nuanced, context-sensitive responses. It is important to note that these interpretations are offered as informed hypotheses rather than definitive conclusions.

With regard to DeepSeek, regardless of its more favorable DISCERN and EQIP scores (58 and 80, respectively, in the repetition mode), the observed variability in outputs when the same prompt is repeatedly entered raises an additional concern. Patients who ask the same question repeatedly for clarification may receive substantially different responses, potentially leading to confusion or diminished trust. Therefore, in clinical practice, both clinicians and patients should take the following two points into consideration: For information generated by ChatGPT, clinicians should remain alert to potential gaps in therapeutic depth and may need to supplement responses with more detailed plan explanations and clinical rationale. For information generated by DeepSeek, clinicians must recognize that response consistency cannot be guaranteed; if patients report receiving contradictory information, verification of key points is recommended. For the two models, the lack of transparency regarding information sources underscores the importance of clinicians as interpreters and validators of AI-generated content. Patients should be encouraged to bring AI-generated information to clinical consultations for discussion, rather than acting on it independently.

When queries involve rare but severe symptom combinations, atypical treatment responses, or emerging therapies, both models may provide incomplete or overly generalized information due to underrepresentation of their training data—a common limitation of current AI tools. For “edge case” queries that fall outside typical clinical presentations, involve multiple interacting variables, or lie within areas of medical uncertainty, AI systems face their greatest challenges. These are the scenarios where AI-assisted tools most require clinical supervision. Accordingly, for complex queries with vague symptom descriptions or references to multiple concurrent chronic conditions, model responses should be treated as preliminary clues rather than definitive diagnoses. However, optimizing prompt design may serve as an important strategy for enhancing the quality, reliability, and clinical utility of AI-generated medical content. For example, a study on prostate cancer suggests that using full-sentence prompts may contribute to improving content quality (30). Our findings resonate with the educational concerns raised by Sarikahya et al. (50). In their qualitative study of nursing faculty experiences, they noted that while ChatGPT could facilitate access to information, its outputs carried a risk of undermining critical thinking and must be deliberately embedded within human-supervised educational frameworks. This parallel strengthens our central argument: regardless of how readable or structurally sound AI-generated medical information may be, it should always be reviewed and contextualized by qualified healthcare professionals before being delivered to patients.

In this study, both the repetitive and cyclic query modes were employed to simulate real-world user interactions with AI systems. Nonetheless, there are several limitations to be noted. First, all query terms were derived from Google Trends. Although this approach reflects real-world patient search behavior, the resulting list may not capture the full range of patient information needs. Future research should therefore complement trend-based selection with qualitative data obtained from patient interviews or analyses of online patient communities. Additionally, some terms may exhibit semantic overlap in medical contexts. Future studies can employ more refined semantic analysis tools, such as Google Correlate, topic modeling, or clinical terminology standardization systems, to cluster and deduplicate query terms, thereby constructing a more discriminative question set. Furthermore, the selected queries were based on global data and may not fully reflect region-specific concerns or cultural nuances in information-seeking behaviors. Second, to control variables, standardized, keyword-style prompts directly converted from search terms were utilized. This method may differ from real-world clinician-patient interactions, which often involve more complex, multi-layered inquiries, follow-up questions, or emotionally nuanced expressions. Such simplification may overestimate model performance in authentic, unstructured conversational settings. Future research should adopt query methods that more closely mimic natural user behavior to validate the present findings. We advocate for more ecologically valid study designs—for example, allowing real patients or lay volunteers to interact with AI systems through natural, multi-term conversations. Such designs would enable researchers to assess not only the readability and quality of the final outputs but also the interactive process itself, including the model's ability to manage ambiguity, respond empathetically, and sustain conversational flow. Third, this study lacks a systematic, line-by-line verification process grounded in authoritative clinical guidelines (e.g., from FIGO or ACOG). While our assessments of quality and readability relied on multiple established instruments (ARI, FRES, GFI, DISCERN, and EQIP), these tools focus primarily on structural quality, readability, and transparency; they are not intended to verify each medical claim against current clinical standards. Although the clinical expertise of our assessors added meaningful value to the evaluation, it cannot replace a structured, guideline-based validation process. We therefore call for future research to integrate readability and structural quality metrics with rigorous fact-checking procedures developed in collaboration with clinical experts. Fourth, the expert evaluators were experienced and blinded to the model sources, however, they were all drawn from the healthcare system of a single country. This may introduce cultural or clinical practice biases, potentially affecting the generalizability of the quality assessment results. We recommend that future research involve a transnational expert panel comprising evaluators from different healthcare systems, cultural backgrounds, and patient populations. Fifth, as a cross-sectional evaluation of model outputs, this study reveals potential trends and differences but has limited capacity for causal inference. Model performance may also evolve with updates and changes in prompt engineering strategies. Finally, while the readability metrics employed have been widely accepted, they are formula-based and may not fully capture all dimensions of text comprehensibility across diverse patient populations. Future research should incorporate direct patient testing to validate these findings with target audiences.

Based on this exploratory study, future research could delve into the several directions. First, directly engaging patients with varying levels of health literacy in testing AI-generated information would provide more definitive evidence regarding its comprehensibility and usability, while also offering insights into how patients incorporate such information into real-world decision-making. Second, conducting specialized studies on the aforementioned “edge cases” would help clarify the boundaries for safe AI use and inform the development of targeted protective measures. Third, examining whether structured AI usage guidelines for clinicians and patients can improve clinical outcomes or patient experience would generate empirical evidence to optimize integration strategies. Fourth, expanding the evaluation scope to include non-English query scenarios and diverse healthcare settings can test the generalizability of current findings and identify context-specific considerations. Fifth, long-term, iterative assessments are needed to track model performance across updates, enabling timely identification of emerging strengths or weaknesses. Finally, research into prompt engineering strategies, fine-tuning methods, or dedicated simplified models holds promise for identifying practical pathways to enhance readability and quality without compromising accuracy.

## 5 Conclusion

This study comparatively analyzes two large language models, ChatGPT and DeepSeek, in generating patient-oriented information on adenomyosis. The findings indicate that while DeepSeek demonstrates superior performance in readability and several information quality metrics, ChatGPT exhibits greater consistency in output stability. More importantly, texts generated by both models exceed the recommended readability level for public health information, a shared practical limitation that directly affects patient comprehension. For clinicians, these findings underscore the importance of proactive engagement with AI-generated information that patients may bring into consultations, necessitating a role of such content as interpreters, verifiers, and personalizers. For patients, AI tools may serve as an effective starting point for understanding their condition but should be viewed as a supplement to, rather than a replacement for, professional medical advice. With the increasing integration of AI tools into healthcare systems, the focus should shift from technical comparisons toward understanding of their practical role in clinical workflows and patient care pathways. By translating technical findings into actionable recommendations for key stakeholders—who will ultimately determine whether AI can fulfill its potential in supporting gynecological health information dissemination and shared decision-making—this study contributes to advancing the shift.

## Data Availability

The data used in this study were obtained from Google Trends (https://www.google.com/trends). The raw search trend data were retrieved from January 1, 2004, to September 30, 2025. All figures in this manuscript were generated by the authors based on the retrieved raw data. The datasets generated and analyzed during the study period are available from the corresponding author upon reasonable request.
