# Supplementary material for: Evaluating the quality and readability of AI-generated information on adenomyosis: a comparative analysis of ChatGPT and deepseek regarding query-model consistency
Source: Front Public Health. 2026 Jun 10;14:1785229. doi: 10.3389/fpubh.2026.1785229 (PMC13291018; doi:10.3389/fpubh.2026.1785229)
Supplement: Supplementary file 1 [file Supplementary_file_1.docx]

**Table S1.Accuracy-Related Scores for Both Models**

| Instrument Item | DeepSeek (Mean ± SD) | ChatGPT (Mean ± SD) | *p*-value^1^ |
| --- | --- | --- | --- |
| **DISCERN Item 9** (Benefits description) | 5±0 | 4.36±1.41 | <0.001 |
| **DISCERN Item 10** (Risks description) | 4.84±0.73 | 3.76±1.33 | <0.001 |
| **DISCERN Item 11** (No treatment consequences) | 3.88±1.13 | 2.40±1.08 | 0.852 |
| **DISCERN Item 12** (Quality of life impact) | 4.28±0.89 | 3.20±0.95 | 0.775 |
| **DISCERN Item 13** (Multiple treatment options) | 4.84±0.47 | 4.40±1.32 | 0.003 |
| **DISCERN Item 14** (Shared decision-making support) | 4.84±0.80 | 3.40±1.29 | <0.001 |
| **DISCERN Item 15** (Treatment choice clarity) | 4.76±0.52 | 4.0±1.35 | 0.006 |
| **EQIP Item 6** (Medical accuracy) | 1±0 | 0.92±0.27 | 0.003 |

^1^：Welch Two Sample t-test

**Based on the statistical data provided in Table S1, we can clearly observe performance differences between the two models across multiple dimensions related to "accuracy." These differences are not only statistical (as indicated by p-values) but also hold significant clinical and communication implications.**

1. Foundational Medical Accuracy (EQIP Item 6)

The data show that DeepSeek achieved a perfect score (1.00 ± 0) in foundational medical accuracy, significantly higher than ChatGPT (0.92 ± 0.27), with p = 0.003. When generating responses, DeepSeek demonstrates greater precision in handling foundational medical facts (such as definitions, etiology, and symptom descriptions), with almost no occurrence of misinformation. While ChatGPT's score is also high, its score fluctuation (standard deviation 0.27) suggests instances where its facts were less precise or its phrasing ambiguous in some responses. This directly addresses your concern: under the prerequisite of "information being accurate and error-free," DeepSeek demonstrates higher reliability within the context of this study.

2. Precision and Balance of Treatment Information (DISCERN Items 9, 10, 13, 14, 15)

The analysis reveals that DeepSeek demonstrates significant advantages in the following areas: In describing benefits (Item 9): DeepSeek (5.00 ± 0) significantly outperforms ChatGPT (4.36 ± 1.41), p < 0.001. DeepSeek can articulate the advantages of various treatment options more clearly and comprehensively. In describing risks (Item 10): DeepSeek (4.84 ± 0.73) significantly outperforms ChatGPT (3.76 ± 1.33), p < 0.001. This is a particularly critical finding. Accurately conveying risks is central to the ethics of medical information. DeepSeek not only mentions risks but describes them more specifically and quantitatively. ChatGPT's lower and highly variable score in this item indicates a significant weakness in risk communication, sometimes potentially omitting or downplaying important treatment risks. In presenting multiple treatment choices (Item 13): DeepSeek (4.84 ± 0.47) significantly outperforms ChatGPT (4.40 ± 1.32), p = 0.003. DeepSeek shows a greater tendency to present a comprehensive spectrum of treatment options rather than being limited to a single choice. In supporting shared decision-making (Item 14): DeepSeek (4.84 ± 0.80) significantly outperforms ChatGPT (3.40 ± 1.29), p < 0.001. This indicates that DeepSeek's responses, in both structure and content, are better at empowering patients to engage in discussions with their doctors based on their own values, representing a more advanced manifestation of "precise" information.

3. Handling Uncertainty and Describing Consequences (DISCERN Items 11, 12)

Although no statistically significant differences were observed, it is noteworthy that both models scored at a moderate level in describing "consequences of no treatment" (Item 11) and "impact on quality of life" (Item 12). This reveals a common challenge for AI in providing prospective prognostic information and handling medical uncertainty.

**Item-Level Analysis of DISCERN and EQIP**

**DISCERN Items 9-15: These items assess the quality of information on treatment choices, including:**

Item 9. Does it describe how each treatment works?

Item 10. Does it describe the benefits of each treatment?

Item 11. Does it describe the risks of each treatment?

Item 12. Does it describe what would happen if no treatment is used?

Item 13. Does it describe how the treatment choices affect overall quality of life?

Item 14. Is it clear that there may be more than one possible treatment choice?

Item 15. Does it provide support for shared decision-making? Does the information help patients make supportive decisions based on their own values and preferences?

EQIP Section 6 : “Accuracy: Is the information medically accurate? (This may require expert knowledge to judge.)”
